# Validation of Prognostic Scores in Extracorporeal Life Support: A Multi-Centric Retrospective Study

**DOI:** 10.3390/membranes11020084

**Published:** 2021-01-24

**Authors:** Christoph Fisser, Luis Alberto Rincon-Gutierrez, Tone Bull Enger, Fabio Silvio Taccone, Lars Mikael Broman, Mirko Belliato, Leda Nobile, Federico Pappalardo, Maximilian V. Malfertheiner

**Affiliations:** 1 Department of Internal Medicine II, University Hospital Regensburg, 93053 Regensburg, Germany; maximilian.malfertheiner@ukr.de; 2 Department of Intensive Care Medicine, Erasme Hospital, Université Libre de Bruxelles, Route de Lennik, 808, B-1070 Brussels, Belgium; doctor.rincon@gmail.com (L.A.R.-G.); ftaccone@ulb.ac.be (F.S.T.); leda.nobile@gmail.com (L.N.); 3 Clinic of Cardiology, St. Olavs University Hospital, 7030 Trondheim, Norway; tonebullenger@gmail.com; 4 ECMO Centre Karolinska, Karolinska University Hospital, 171 64 Stockholm, Sweden; lars.broman@sll.se; 5 Department of Physiology and Pharmacology, Karolinska Institutet, 171 77 Stockholm, Sweden; 6 UOS Advanced Respiratory Intensive Care Unit, UOC Anestesia e Rianimazione 1, Fondazione I.R.C.C.S. Policlinico San Matteo, 27100 Pavia, Italy; m.belliato@gmail.com; 7 San Raffaele Scientific Institute, Vita Salute University, 20132 Milan, Italy; fedepappa.71@gmail.com; 8 Department of Anesthesia and Intensive Care, IRCCS ISMETT, UPMC Italy, 90133 Palermo, Italy

**Keywords:** ECMO, score, RESP score, SAVE score, validation, ECLS

## Abstract

Multiple prognostic scores have been developed for both veno-arterial (VA) and veno-venous (VV) extracorporeal membrane oxygenation (ECMO), mostly in single-center cohorts. The aim of this study was to compare and validate different prediction scores in a large multicenter ECMO-population. Methods: Data from five ECMO centers included 300 patients on VA and 329 on VV ECMO support (March 2008 to November 2016). Different prognostic scores were compared between survivors and non-survivors: APACHE II, SOFA, SAPS II in all patients; SAVE, modified SAVE and MELD-XI in VA ECMO; RESP, PRESET, ROCH and PRESERVE in VV ECMO. Model performance was compared using receiver-operating-curve analysis and assessment of model calibration. Survival was assessed at intensive care unit discharge. Results: The main indication for VA ECMO was cardiogenic shock; overall survival was 51%. ICU survivors had higher Glasgow Coma Scale scores and pH, required cardiopulmonary resuscitation (CPR) less frequently, had lower lactate levels and shorter ventilation time pre-ECMO at baseline. The best discrimination between survivors and non-survivors was observed with the SAPS II score (area under the curve [AUC] of 0.73 (95% CI 0.67–0.78)). The main indication for VV ECMO was pneumonia; overall survival was 60%. Lower PaCO_2_, higher pH, lower lactate and lesser need for CPR were observed among survivors. The best discrimination between survivors and non-survivors was observed with the PRESET score (AUC 0.66 (95% CI 0.60–0.72)). Conclusion: The prognostic performance of most scores was moderate in ECMO patients. The use of such scores to decide about ECMO implementation in potential candidates should be discouraged.

## 1. Introduction

Multiorgan failure (MOF) is a common complication in critically ill patients requiring intensive care unit (ICU) admission and is associated with a high mortality rate. Therefore, multiple scoring systems such as the sepsis-related organ failure assessment (SOFA) [1], the simplified acute physiology score (SAPS II) [2] and the acute physiology and chronic health evaluation score (APACHE II) [3] have been developed to quantify the severity of illness, to understand the evolution of the acute illness, to evaluate the impact of treatment and to predict outcome in critically ill patients [1,2,3]. Due to the rapid progression in therapeutic options for such patients, prognostic scores have also been developed for those undergoing extracorporeal membrane oxygenation (ECMO) to eventually allocate expensive and complex resources.

According to the Extracorporeal Life Support Organization (ELSO) [4], indications for veno-arterial (VA) and veno-venous (VV) are severe refractory cardiogenic shock and respiratory failure with an expected mortality risk above 50%, respectively. However, these indications are still controversial and differ among centers. Therefore, scoring systems might be helpful to identify subgroups of patients in whom the initiation of ECMO would be very beneficial or associated with a very low likelihood of survival.

For VA ECMO patients, the SAVE score [5], the modified SAVE score (with addition of lactate) [6] and the MELD-XI [7] are largely used; and in VV ECMO patients, the RESP score [8], the PRESERVE score [9], the ROCH score [10] and the PRESET [11] are reportedly used to predict outcome and to guide decision-making for whom to support with ECMO; this would be beneficial in cases of limited resources such as the COVID-19 pandemic in order to enable better allocation. Most scores are derived from small single-center cohorts [6,9,10,11] and have not been validated in large multicenter cohorts.

In our study we compared specific ECMO scores with general ICU scores in a large multicenter cohort of patients from five European high-volume ECMO centers and analyzed which scores performed most accurately in the two most used ECMO modes.

## 2. Material & Methods

### 2.1. Study Population

Consecutive patients with severe ARDS or cardiogenic shock requiring ECMO either in VV or VA mode between March 2008 to November 2016 were included from five European high-volume ECMO centers (Brussels, Milan, Stockholm, Pavia, and Regensburg). Patients <18 years and with configurations other than VV or VA were excluded. The requirement of individual patient consent and necessity of approval for the data report complied with the declaration of Helsinki and were waived by the local ethics committee because of the study’s design and data collection from routine care.

Indications for ECMO were based on local ECMO protocols and ELSO guidelines [4]. Contraindications were in agreement with ELSO guidelines [4] such as advanced age, chronic irreversible organ dysfunction, malignancies with fatal prognosis within 1 year, and contraindication for therapeutic anticoagulation.

### 2.2. Data Collection

Routine data (e.g., demographics, diagnosis group, biochemistry, cardiac and respiratory parameters) were assessed before ECMO initiation and were extracted from the electronic patient data management systems. Survival was assessed at ICU discharge.

The following scores assessing the severity of illness were applied to both the VA and VV cohorts: APACHE II [3], SOFA [1] and SAPS II [2]. Additionally, specific ECMO scores such as SAVE [5], modified SAVE [6], and MELD-XI [7] scores were assessed in the VA cohort, whereas RESP [8], PRESET [11], PRESERVE [9], and ROCH [10] score were evaluated in the VV cohort. More details of each score are presented in Supplemental Tables S1–S10. Only patients with a complete data set were included in the analysis. The primary objective of this retrospective multicenter study was to compare ECMO-specific scores with general ICU scores and to predict mortality in VA and VV ECMO. Secondary outcome included the identification of the most accurate predictive score for each subgroup of patients.

### 2.3. Statistical Analyses

Unless otherwise indicated, descriptive data were expressed as medians and interquartile range (IQR) or as frequencies (%) of each category. The subgroups of patients (survivors and non-survivors) were compared using the Chi-square test for categorical variables and the Mann–Whitney U test for continuous variables. Scores were retrospectively calculated according to original publications [1,2,3,5,6,7,9,10,11]. In order to assess discrimination and calibration, each score was put as a test variable with mortality (no/yes) as the outcome variable in a univariate logistic regression analysis. Discrimination was assessed by area under the receiver-operating characteristics curve (AUC), where an AUC of 0.50 suggests no discrimination, 0.50 to 0.69 considered moderate, 0.70 to 0.79 acceptable, 0.80 to 0.89 excellent, and more than 0.9 as outstanding [12]. AUC was compared using an algorithm suggested by DeLong et al. [13]. Calibration was assessed with a Hosmer–Lemeshow (HL) test and visually by calibration plots using the module pmcalplot in Stata [14]. Model comparison also included calculation of Akaike and Bayesian Information Criterions (AIC and BIC, respectively), which are used to assess model fit while penalizing the number of estimated parameters. The model with the lowest AIC and BIC score was preferred. A two-sided *p*-value < 0.05 was considered a statistically significant difference. Data analyses were performed with the software package Stata (v.16.0, StataCorp, 4905 Lakeway Drive, College Station, TX 77845, USA).

## 3. Results

A total of 629 ECMO patients were included in this study; 300 in the VA and 329 in the VV ECMO cohort.

### 3.1. VA ECMO Population

The cohort consisted mainly of men (66.3%) with a median age of 57 years (Table 1). The main indication for VA ECMO was cardiogenic shock (53%), septic shock (20%), and refractory cardiac arrest (19%). A total of 153 (51%) patients survived to ICU discharge. Pre-ECMO cardiac arrest and mechanical ventilation > 7 days were observed less frequently in survivors than in non-survivors (26% vs. 50%, *p* < 0.001; 40% vs. 51%, *p* = 0.045), respectively. Blood gas analysis before ECMO initiation revealed lower levels of lactate and higher levels of bicarbonate and pH among survivors (Table 1).

**Figure 1:**
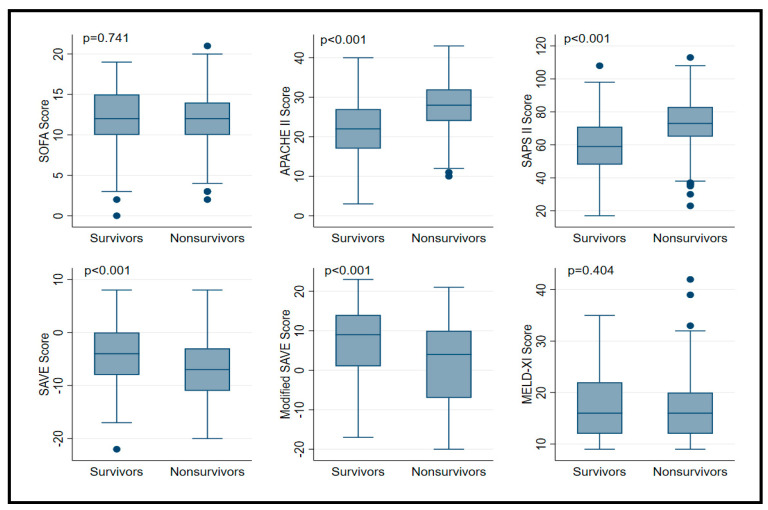
Boxplot of prognostic score values in in patients supported with veno-arterial extracorporeal membrane oxygenation according to survivors and non-survivors. Data are expressed as median, minimum, maximum, 25. percentile, and 75. percentile.

**Figure 2:**
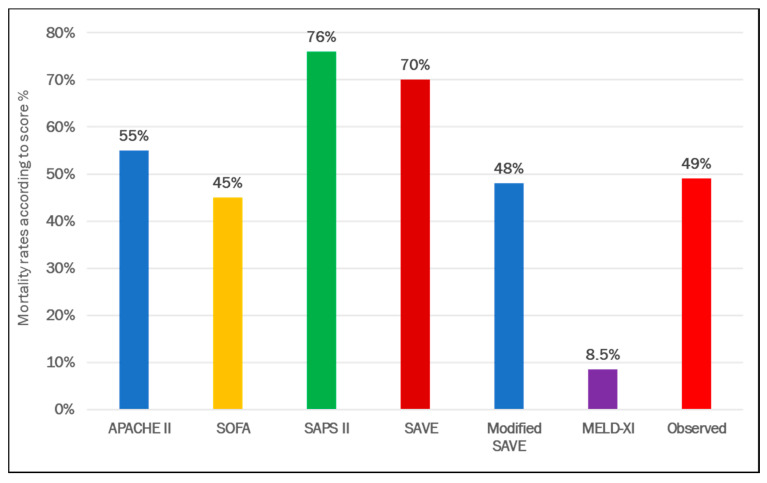
Comparison of predicted and observed mortality rates in patients supported with veno-arterial extracorporeal membrane oxygenation.

**Figure 3:**
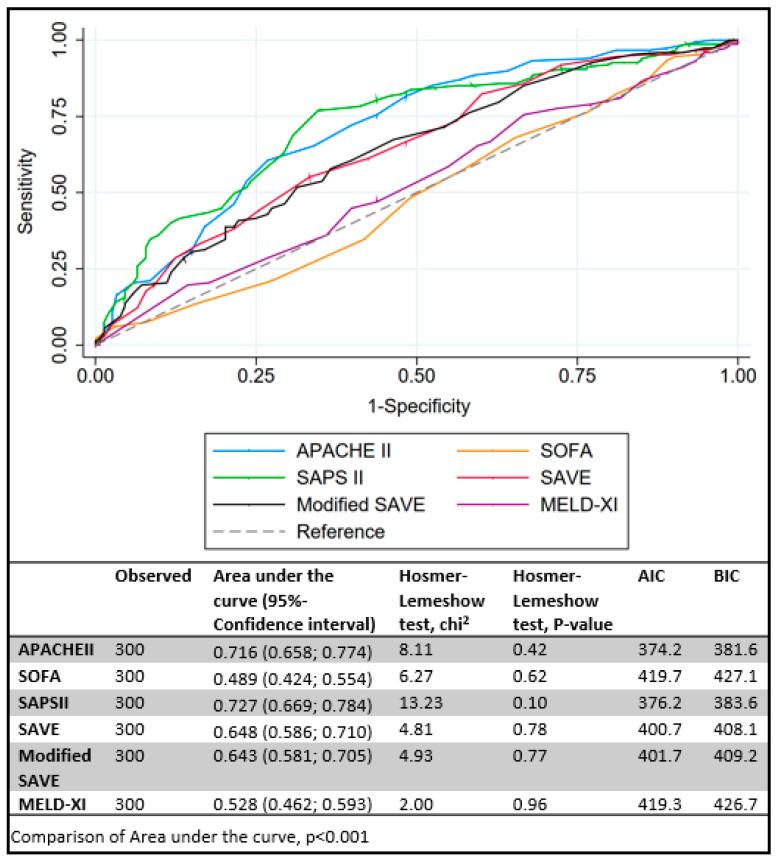
Comparison of area under the receiver-operating characteristics curve (AUC) for (A) veno-arterial extracorporeal membrane oxygenation scores. Akaike and Bayesian Information Criterions (AIC and BIC, respectively).

Predictive scores for VA ECMO are presented in Table 2. APACHE II, SAPS II, SAVE and modified SAVE score, but not MELD-XI and SOFA were significantly different between survivors and non-survivors (Figure 1). Expected mortality rates were quite different between scores, ranging from 8.5 to 76%. Compared to observed mortality rate, the greatest amount of overestimation was observed with SAPS II and SAVE scores (Figure 2). Best discrimination for ICU survival was offered by SAPS II and APACHE II score (AUC = 0.727 (95% CI: 0.669 to 0.784); AUC = 0.716 (95% CI: 0.658 to 0.774)) with good calibration (HL Chi^2^ statistic of 13.23 (*p* = 0.10) and 8.11 (*p* = 0.42)). Other scores, such as SOFA, SAVE, modified SAVE, and MELD-XI performed less accurately (Figure 3). Calibration plots for each score are depicted in Figure S1. APACHE II showed best calibration, SAVE and SAPS II deviated in calibration for extreme scores. Poor calibration was observed for MELD-XI and SOFA (Figure S1).

### 3.2. VV ECMO Population

The median age in the VV ECMO study population was 53 years, 67.2% were males. Most patients suffered from bacterial pneumonia (41.9%) or viral pneumonia (19.1%). A total of 197 (60%) patients survived to ICU discharge. Survivors had a significantly higher platelet count and less frequently required cardiopulmonary resuscitation before ECMO (Table 3). Positive end expiratory pressure was higher in survivors (14 cm H_2_O (IQR: 10–16) vs. non-survivors: 12 cm H_2_O (IQR: 9–15), *p* = 0.005). Survivors had lower lactate and pCO_2_ and higher pH (Table 3).

**Figure 4:**
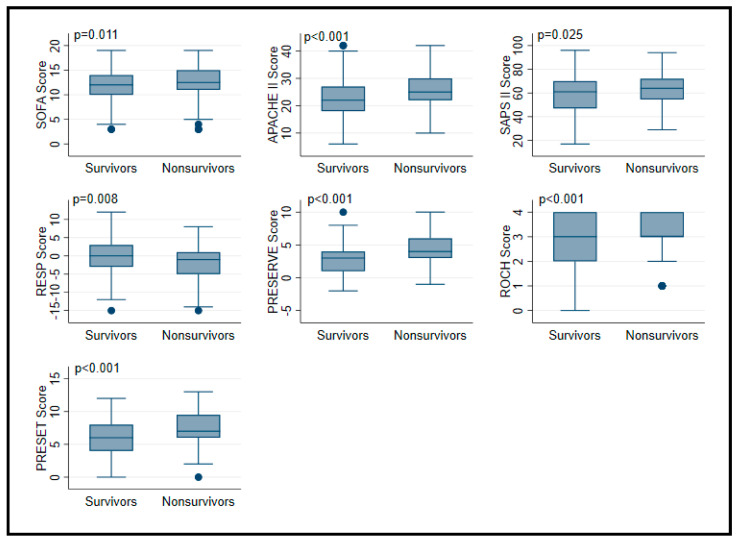
Boxplot of prognostic score values in in patients supported with veno-venous extracorporeal membrane oxygenation according to survivors and non-survivors. Data are expressed as median, minimum, maximum, 25. percentile, and 75. percentile.

**Figure 5:**
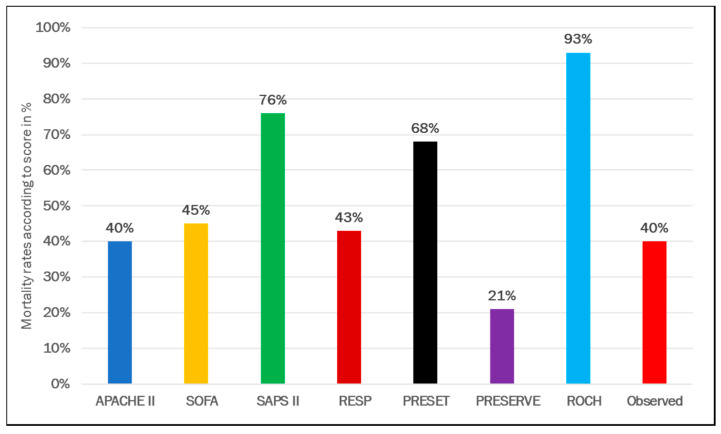
Comparison of predicted and observed mortality rates in patients supported with veno-venous extracorporeal membrane oxygenation.

**Figure 6:**
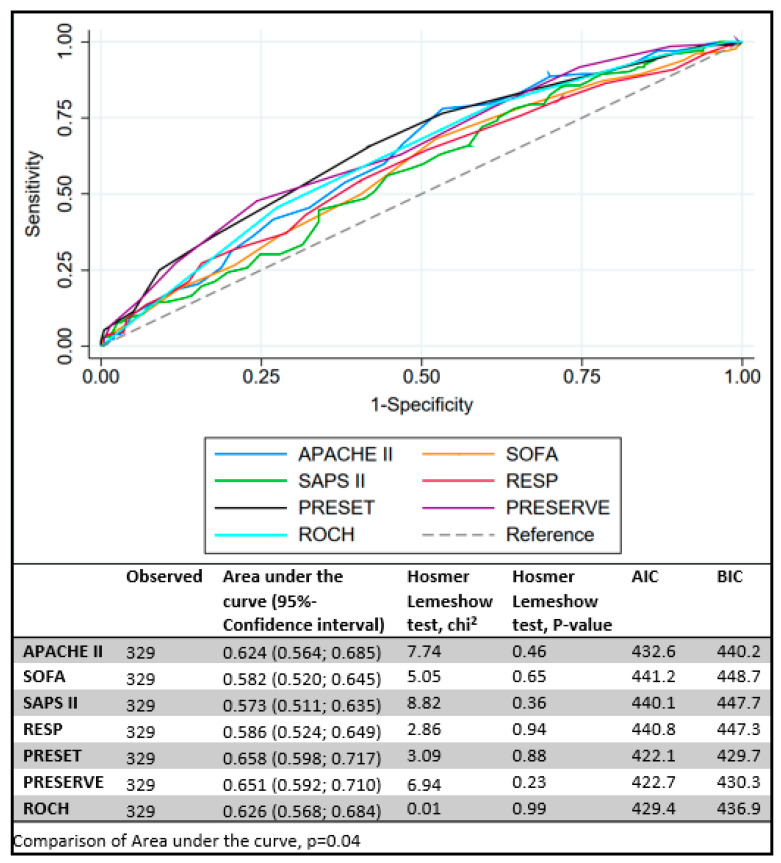
Comparison of area under the receiver-operating characteristics curve (AUC) for veno-venous extracorporeal membrane oxygenation scores. Akaike and Bayesian Information Criterions (AIC and BIC, respectively).

All tested scores (SOFA, APACHE II, SAPS II, RESP, PRESERVE, ROCH, PRESET) were significantly different between survivors and non-survivors (Table 4, Figure 4). Predicted mortality rate was higher using SAPS, PRESET, and ROCH scores, whereas PRESERVE score predicted underpredicted mortality compared to observed (Figure 5). Similar AUCs were observed between all applied scores (Figure 6). PRESET and PRESERVE scores performed best, showing moderate discrimination (AUC = 0.658 (95% CI: 0.598–0.717); AUC = 0.651 (95% CI: 0.592–0.710), and modestly good calibration (HL Chi^2^ statistic of 3.09 (*p* = 0.88) and 6.94 (*p* = 0.23)). Good calibration was observed with PRESERVE, PRESET, ROCH, and RESP score (Figure S2).

## 4. Discussion

This study provides new insights in the validation of established general ICU and dedicated ECMO scores in a large-scale mixed cohort of patients supported with either VA or VV ECMO from five high-volume European ECMO centers. In total, 629 ECMO patients were included and analyzed.

Survivors on VA support were younger, had higher GCS, higher pH, and lower levels of lactate, and were less often ventilated >7 days compared to non-survivors. Expected mortality rates between scores for this patient cohort were overestimated with APACHE II, SAPS II, SAVE, and underestimated with SOFA, modified SAVE, and MELD-XI. General ICU scores such as APACHE II and SAPS II best discriminated survivors from non-survivors. The specific ECMO scores, SAVE, and modified SAVE were inferior. SOFA score performed the worst.

Similar results were seen in the VV cohort. Expected and observed mortality rates were best matched by the APACHE II, SOFA, and RESP score. However, the absolute values only partly reflected AUC values, in which PRESET score discriminated best, although suboptimally.

Overall, general ICU scores were superior in the VA cohort as compared to those devised for ECMO. This did not apply for the VV cohort. These differences might stem from the fact that general ICU scores include more variables reflecting cardiac than respiratory parameters [2,3].

The number of included variables differed between scores (Table S1). General ICU scores such as APACHE II [3] and SAPS II [2] consist of 15 and 17 variables, respectively, whereas VA ECMO scores are composed of 9 or 10 variables [5,6]. Similar is true for VV ECMO scores (using up to 10) [8,9,10,11]. SOFA score is in the middle with six variables, however, SOFA performed the worst in the VA cohort.

In general, scores performed worse in the current study than in the score derivation studies [1,2,3,5,6,7,8,9,10,11] and none performed exceptionally well [12]. In contrast to previous studies based on register data [5,8] with, e.g., full physiologic data of only 23% in the SAVE study [5], this analysis represents data from complete datasets only. Therefore, these two European cohorts (VV and VA) challenge the performance of the scores in a heterogenous population. Unfortunately, the discrimination between survivors and non-survivors was moderate at best. In the current study, a large discrepancy between the predicted and the observed mortality was up to 41% in the VA and up to 53% in the VV. One can argue that for the general ICU scores these mismatches might relate to the different patient populations (e.g., septic patient cohort for the compilation of SOFA score [1]). However, for the specific ECMO scores, similar observations were seen in the current analysis. Thus, the clinician might incorporate the comparison of his own patient population with the studied population, respectively, to guide further management.

Primary endpoints differed between studies and ranged from ICU mortality to survival at 6 months [1,9]. For the current analysis, we chose successful discharge from ICU because this value can be easily assessed without any nonresponse bias. The PRESERVE study chose survival at 6 months post-ICU discharge and therefore our data might be limited when applied to this score. However, ICU survival in the current cohort was even lower than the predicted 6-months survival by the PRESERVE score.

The simple scores might be less inaccurate, while more complex scores may be difficult to use for the bedside clinician. However, due to improvement in technologies, most of the scores can be automatically calculated by patient data management systems at bedside. The current validation study on a large ECMO cohort reflects clinical day-to-day routine: scores might be helpful, but only a piece of the complex puzzle of a critically ill patient, made by a bundle of several therapeutic issues. Therefore, a clinical decision should not rely solely on risk scores, but be incorporated in the complex interaction of clinical status, experience, clinical studies, patients’ wishes, as well as variables not evaluated in these scores such as frailty [15]. Indeed, in the ICU, it is hard to mirror patient status with only 3–17 score parameters; however, until further evidence is provided in intensive care, we have to find a compromise between evidence and eminence-based practice until we can further translate patient status into absolute score numbers.

### Limitations

A direct causal relationship cannot be inferred due to the retrospective study design. The participating units reflect highly experienced ECMO centers. Therefore, the results might not be generalizable. However, due to the multicenter approach, differences might be harmonized. Survival was defined as successful discharge from ICU in contrast to some of the derivation studies [2,3,5,6,7,8,9,10]. However, the observed mortality rates in the current analysis were higher than expected according to the predicted mortality rate of many of the derivation studies. Comparison between centers was not performed since the aim was to apply the scores in a large-scaled multicenter cohort. ENCOURAGE score [16] was not assessed due to missing values. In contrast to other studies [5,8], the current data was not derived from registries, which should be considered a strength. Only complete patient data sets were included in the analysis and the data generated from five independent centers likely eliminate single-center specifics and increase the potential of result generalizability. Further prospective studies are needed.

## 5. Conclusions

The performance of most risk scores was suboptimal in patients on VV and VA ECMO. In VA ECMO patients, best discrimination between survivors and non-survivors was seen using non-ECMO scores, whereas in VV, PRESET score performed best. The use of such scores to decide about ECMO implementation in potential candidates should be discouraged.

## Figures and Tables

**Table 1:** Patient characteristics on patients on extracorporeal membrane oxygenation (ECMO) before implantation according to survival (veno-arterial ECMO cohort).

	VA Total (*n* = 300)	VA Survivors (*n* = 153)	VA Non-Survivors (*n* = 147)	*p*-Value
Demographics, vital signs & laboratory chemistry				
Age in years	57 (46–65)	56 (42–63)	58 (47–65)	0.05
Weight in kg	77 (70–90)	78 (68–89)	77 (70–90)	0.946
Female sex	101 (33.7%)	53 (34.6%)	48 (32.7%)	0.72
Heart rate/min	101 (82–120)	106 (87–125)	96 (80–119)	0.059
Mean arterial pressure in mmHg	68 (57–80)	70 (57–80)	66 (56–80)	0.345
Glasgow Coma Scale	3 (3–13)	9 (3–15)	3 (3–3)	**<0.001**
Acute renal failure	160 (53.3%)	76 (49.7%)	84 (57.1%)	0.195
Creatinine in mg/dL	1.50 (1.04–2.18)	1.50 (1.10–2.32)	1.50 (1.00–2.07)	0.66
Temperature in °C	36.5 (35.3–37.2)	36.6 (35.5–37.4)	36.4 (34.8–37.1)	**0.025**
Leucocytes/nL	11.7 (7.9–16.9)	11.8 (8.1–18.4)	10.8 (7.5–16.0)	0.116
Platelets × 10^3^/µL	131.5 (83.0–196.8)	125.0 (82.0–193.0)	141.0 (83.0–205.0)	0.695
Cardiopulmonary resuscitation pre ECMO	113 (37.7%)	39 (25.5%)	74 (50.3%)	**<0.001**
Mechanical ventilation pre-ECMO for >7 days	136 (45.3%)	78 (51.0%)	58 (39.5%)	**0.045**
Mechanical ventilatory settings				
Inspiratory pressure in cmH_2_O	24 (20–32)	25 (20–33)	23 (20–32)	0.20
Blood gas analysis				
pH	7.27 (6.70–7.69)	7.30 (6.80–7.69)	7.23 (6.70 –7.57)	**<0.001**
PaO_2_ in mmHg	77 (63–108)	76 (64–100)	80 (62–112)	0.30
PaO_2_/FiO_2_ in mmHg	105 (67–193)	100 (67–190)	113 (67–202)	0.34
Bicarbonate in mmoL/L	19 (14.2–22.1)	20.4 (16.0–23.2)	18 (13.0–21.1)	**<0.001**
Lactate in mmoL/L	5.3 (2.3–10.5)	4.4 (1.9–8.4)	7.1 (3.0–11.5)	**0.001**

Data are expressed as *n* (%), or median (interquartile range); significant *p* values (*p* < 0.05) marked in bold.

**Table 2:** Scoring results in patients on ECMO before implantation according to survival (veno-arterial ECMO cohort).

	VA Total (*n* = 300)	VA Survivors (*n* = 153, 51%)	VA Non-Survivors (*n* = 147, 49%)	*p*-Value
Prediction scores				
SOFA ^a^	12 (10–14)	12 (10–15)	12 (10–14)	0.74
APACHE II ^b^	25 (19–30)	22 (17–27)	28 (24–32)	**<0.001**
SAPS II ^b^	67 (55–78)	59 (48–71)	73 (65–83)	**<0.001**
SAVE ^c^	−6 (−9 to −2)	−4 (−8 to 0)	−7 (−11 to −3)	**<0.001**
Modified SAVE ^c^	7.5 (−4–12)	9 (1–14)	4 (−7–10)	**<0.001**
MELD-XI ^d^	16 (12–22)	16 (12–22)	16 (12–20)	0.40

Data are expressed as *n* (%), or median (interquartile range); significant *p* values (*p* < 0.05) marked in bold. ^a^ ICU mortality; ^b^ In-hospital mortality; ^c^ survival to hospital discharge; ^d^ 90-day mortality.

**Table 3:** Patient characteristics on patients on ECMO before implantation according to survival (veno-venous ECMO cohort).

	VV Total (*n* = 329)	VV Survivors (*n* = 197)	VV Non-Survivors (*n* = 132)	*p*-Value
Demographics, vital signs & laboratory chemistry				
Age in years	53 (41–63)	50 (39–62)	56 (45–63)	**0.005**
Weight in kg	80 (70–92)	80 (70–98)	80 (69–90)	0.065
Female sex	108 (32.8%)	64 (32.5%)	44 (33.3%)	0.87
Heart rate/min	105 (90–122.8)	105 (90–122)	110 (94–125)	0.258
Mean arterial pressure in mmHg	71 (64–81)	72 (63–84)	68 (62–80)	0.104
Glasgow Coma Scale	3 (3–12)	3 (3–13)	3 (3–11)	0.074
Acute renal failure	112 (34.0%)	59 (29.9%)	53 (40.2%)	0.056
Creatinine in mg/dL	1.15 (0.72–1.91)	1.14 (0.74–1.96)	1.23 (0.70–1.90)	0.905
Temperature in °C	36.9 (36.2–37.5)	37.0 (36.3–37.7)	36.7 (36.0–37.3)	**0.013**
Leucocytes/nL	12.8 (7.8–20.1)	12.7 (7.2–20.0)	13.1 (7.9–20.5)	0.955
Platelets × 10^3^/µL	156.5 (86.3–242.5)	168.0 (109.5–247.5)	138.0 (47.0–236.0)	**0.001**
Cardiopulmonary resuscitation pre ECMO	30 (9.1%)	12 (6.1%)	18 (13.6%)	**0.02**
Mechanical ventilation pre-ECMO for >7 days	89 (27.1%)	46 (23.4%)	43 (32.6%)	0.065
Mechanical ventilatory settings				
Inspiratory pressure in cmH_2_O	35 (31–41)	35 (31–40)	35 (31–41)	0.79
PEEP in cmH_2_O	12 (10–15)	14 (10–16)	12 (9–15)	**0.005**
Plateau pressure in cmH_2_O	32 (29–36)	32 (28–35)	32 (30–36)	0.55
Prone positioning	53 (16.2%)	30 (15.2%)	23 (17.7%)	0.55
Nitric oxide ventilation	55 (16.7%)	34 (17.3%)	21 (15.9%)	0.75
Bicarbonate infusion pre ECMO	46 (14.0%)	26 (13.2%)	20 (15.2%)	0.62
Neuromuscular blockage	234 (71.1%)	134 (68.0%)	100 (75.8%)	0.13
Blood gas analysis				
pH	7.32 (7.22–7.41)	7.35 (7.23–7.42)	7.31 (7.20–7.39)	**0.02**
PaO_2_ in mmHg	62 (53–74)	61 (53–74)	63 (53–74)	0.34
PaO_2_/FiO_2_ in mmHg	68 (55–98)	66 (54–89)	72 (56–107)	0.13
PaCO_2_ in mmHg	54 (43–70)	52 (42–68)	58 (46–72)	**0.03**
Lactate in mmoL/L	2.2 (1.4–4.0)	2.0 (1.4–3.7)	2.6 (1.6–4.5)	**0.01**

Data are expressed as *n* (%), or median (interquartile range); significant *p* values (*p* < 0.05) marked in bold.

**Table 4:** Scoring results in patients on ECMO before implantation according to survival (veno-venous ECMO cohort).

	VV Total (*n* = 329)	VV Survivors (*n* = 197, 60%)	VV Non-Survivors (*n* = 132, 40%)	*p*-Value
SOFA ^a^	12 (10–14)	12 (10–14)	13 (11–15)	**0.011**
APACHE II ^b^	24 (19–28)	22 (18–27)	25 (22–30)	**<0.001**
SAPS II ^b^	62 (51–71)	61 (47–70)	64 (55–72)	**0.025**
RESP ^c^	0 (−4–2)	0 (−3–3)	−1 (−5–1)	**0.008**
PRESERVE ^d^	4 (2–5)	3 (1–4)	4 (3–6)	**<0.001**
ROCH ^b^	3 (2–4)	3 (2–4)	3 (3–4)	**<0.001**
PRESET ^a^	7 (5–9)	6 (4–8)	7 (6–10)	**<0.001**

Data are expressed as *n* (%), or median (interquartile range); significant *p* values (*p* < 0.05) marked in bold. ^a^ ICU mortality; ^b^ In-hospital mortality; ^c^ survival to hospital discharge; ^d^ survival by 6 months post-ICU discharge.

## Data Availability

The database will be available upon reasonable request to the authors.

## Supplementary Materials

The following are available online at https://www.mdpi.com/2077-0375/11/2/84/s1, Figure S1: Comparison of predictive performance for all VA ECMO scores; Figure S2: Comparison of predictive performance for all VV ECMO scores; Table S1: Comparison of general ICU scores and ECMO scores; Table S2: APACHE II score: expected mortality rate according to scoring; Table S3: SOFA score: expected mortality rate according to scoring; Table S4: SAPS II score: expected mortality rate according to scoring; Table S5: RESP score: expected survival rate according to scoring; Table S6: PRESERVE score: survival rate according to scoring; Table S7: Roch score: expected mortality rate according to scoring; Table S8: PRESET score: expected mortality rate according to scoring; Table S9: MELD-XI (Model for End-Stage Liver Disease Excluding INR) score: expected mortality rate according to scoring; Table S10: SAVE score: expected survival rate according to scoring.
