# Supplementary material for: Validation of Prognostic Scores in Extracorporeal Life Support: A Multi-Centric Retrospective Study
Source: Membranes (Basel). 2021 Jan 24;11(2):84. doi: 10.3390/membranes11020084 (PMC7912316; doi:10.3390/membranes11020084)
Supplement: Supplementary file 1 [file membranes-11-00084-s001.pdf]

Supplementary material

This appendix is provided by the authors to give additional information about the research.

Validation of Prognostic Scores in Extracorporeal Life Support: A Multi-Centric Retrospective Study

Christoph Fisser [1,*,†], Luis Alberto Rincon-Gutierrez [2,†], Tone Bull Enger [3], Fabio Silvio Taccone [2], Lars Mikael Broman [4,5], Mirko Belliato [6], Leda Nobile [2], Federico Pappalardo [7,8] and Maximilian V. Malfertheiner [1]

[1] Department of Internal Medicine II, University Hospital Regensburg, 93053 Regensburg, Germany; maximilian.malfertheiner@ukr.de
[2] Department of Intensive Care Medicine, Erasme Hospital, Université Libre de Bruxelles, Route de Lennik, 808, B-1070 Brussels, Belgium; doctor.rincon@gmail.com (L.A.R.-G.); ftaccone@ulb.ac.be (F.S.T.); leda.nobile@gmail.com (L.N.)
[3] Clinic of Cardiology, St. Olavs University Hospital, Trondheim, Norway; tonebullenger@gmail.com
[4] ECMO Centre Karolinska, Karolinska University Hospital, Stockholm, Sweden; lars.broman@sll.se
[5] Department of Physiology and Pharmacology, Karolinska Institutet, Stockholm, Sweden
[6] UOS Advanced Respiratory Intensive Care Unit, UOC Anestesia e Rianimazione 1, Fondazione I.R.C.C.S. Policlinico San Matteo, 27100 Pavia, Italy; m.belliato@gmail.com
[7] San Raffaele Scientific Institute, Vita Salute University, Milan, Italy; fedepappa.71@gmail.com
[8] Department of Anesthesia and Intensive Care, IRCCS ISMETT, UPMC Italy, Palermo, Italy
* Correspondence: Christoph.Fisser@ukr.de; Tel.: +49-941-944-17284
† Both authors contributed equally

Table S1: Comparison of general ICU scores and ECMO scores

Score	APACHE II [1]	SOFA [2]	SAPS II [3]	RESP [4]	PRESERVE [5]	ROCH [6]	PRESET [7]	MELD-XI [8]	SAVE [9]	Modified SAVE [10]
Patient cohort	Mixed ICU cohort	Septic patients	Mixed ICU cohort	Acute respiratory failure	Severe ARDS	ARDS brought to a referral center	ARDS	Cirrhotic veterans	Cardiogenic shock	Cardiac and non-cardiac ICU patients requiring VA ECMO
Derivation cohort (+validation)	5815	1643	8369 (4628)	2355 (140)	140 (51; 289)	85	82 (59)	554 (278)	3846 (161)	154
Endpoint	In-hospital mortality	ICU-mortality	In-hospital mortality	In-hospital survival	Survival by 6-months post-ICU discharge	In-hospital mortality	ICU mortality	90 days mortality	Survival to hospital discharge in VA ECMO patients	Survival to hospital discharge in VA ECMO patients
Range	0 to 71	0 to 24	0 to 163	-22 to 15	-2 to 14	0 to 4	0 to 15	6 to >35	-35 to 17	-35 to 32
Internal validation AUC	0.86	NA	0.88	0.74	0.89	0.80	0.85	0.87	0.68	0.84

External validation AUC	NA	NA	0.86	0.92; 0.81	0.67; 0.69	NA	0.70	0.84	0.90	NA
Amount of included parameters	15	6	17	10	8	3	5	3	9	10
Age	X		X	X	X	X			X	X
Body mass index					X					
Weight									X	X
Temperature	X		X							
Hospital days pre ECMO							X			
Mean arterial pressure	X	X					X			
Systolic blood pressure			X							
Diastolic blood pressure									X	X
Heart rate	X		X							
Etiology of cardiogenic shock									X	X
Cardiac arrest				X					X	X
Respiratory rate	X									
Days of mechanical ventilation				X	X				X	X
Peak inspiratory pressure				X					X	X

Plateau pressure					X					
Positive endexpiratory pressure					X					
Neuromuscular blockade agents				X						
Nitric oxide use				X						
Prone positioning					X					
AaDO2 or pO2	X									
pO2/FiO2		X	X							
pCO2				X						
pH	X						X			
Sodium	X		X							
Potassium	X		X							
Creatinine	X	X						X		
Serum urea			X							
Urinary Output			X							
Hematocrit	X									
White blood count	X		X							
Platelets		X					X			
Bilirubin		X	X					X		
Glasgow coma scale	X	X	X							
Central nervous system dysfunction				X						
Bicarbonate	X		X							

Bicarbonate infusion				X					X	X
Lactate							X			X
Chronic health status	X									
Type of admission			X							
Acquired immunodeficiency syndrome			X							
Immunocompromised				X	X					
Metastatic cancer			X							
Hematologic malignancy			X							
SOFA					X	X				
Influenza						X				
Etiology of liver disease								X		
Acute pre-ECMO organ failure									X	X

NA: not available; AUC: area under the curve; ECMO: extracorporeal membrane oxygenation; SOFA: sequential organ failure assessment; AaDO2: alveolar-arterial difference for O2; included values marked with “X”.

Expected mortality rates according to scores:

Table S2: APACHE II score: expected mortality rate according to scoring

APACHE II Score [1]	Approximated in-hospital mortality rates	
	Nonoperative	Postoperative
0-4	4%	1%
5-9	8%	3%
10-14	15%	7%
15-19	25%	12%
20-24	40%	30%
25-29	55%	35%
30-34	73%	73%
>34	85%	88%

Table S3: SOFA score: expected mortality rate according to scoring

SOFA [2]	ICU Mortality in %
0 to 6	< 10%
7 to 9	15 - 20%
10 to 12	40 - 50%
13 to 14	50 - 60%
15	> 80%
>15 to 24	> 90%

Table S4: SAPS II score: expected mortality rate according to scoring

SAPS II [3]	In-hospital mortality
10	1.0%
20	3.7%
30	10.6%
40	24.7%
50	46.1%
60	68.1%
70	83.8%
80	92.5%
90	96.7%
100	98.5%

Table S5: RESP score: expected survival rate according to scoring

RESP [4]	Risk class	In-hospital survival
≥ 6	I	92%
3 to 5	II	76%

-1 to 2	III	57%
-5 to -2	IV	33%
≤ -6	V	18%

Table S6: PRESERVE score: survival rate according to scoring

PRESERVE [5]	6-months survival
0 to 2	97%
3 to 4	79%
5 to 6	54%
≥ 7	16%

Table S7: Roch score: expected mortality rate according to scoring

<table>
<tr><th>Roch [6]</th><th>In-hospital mortality</th></tr>
<tr><td>0</td><td rowspan="3">40%</td></tr>
<tr><td>1</td></tr>
<tr><td>2</td></tr>
<tr><td>3</td><td rowspan="2">93%</td></tr>
<tr><td>4</td></tr>
</table>

Table S8: PRESET score: expected mortality rate according to scoring

PRESET [7]	Risk class	ICU mortality
0-5	I	26%
6 to 9	II	68%
10 to 15	III	93%

Table S9: MELD-XI (Model for End-Stage Liver Disease Excluding INR) score: expected mortality rate according to scoring

MELD-XI [8]	90 days mortality
6-10	3.4%
11-15	3.0%
16-20	8.5%
21-25	31.0%
26-30	61.3%
31-35	82.4%
>35	95%

Table S10: SAVE score: expected survival rate according to scoring

SAVE Score [9]	Risk class	In-hospital survival
≥5	I	75%
1 to 5	II	58%
-4 to 0	III	42%

-9 to -5	IV	30%
≤ -10	V	18%

Modified SAVE [10]

- Cut-off values not given

Figure S1: Comparison of predictive performance for all VA ECMO scores.

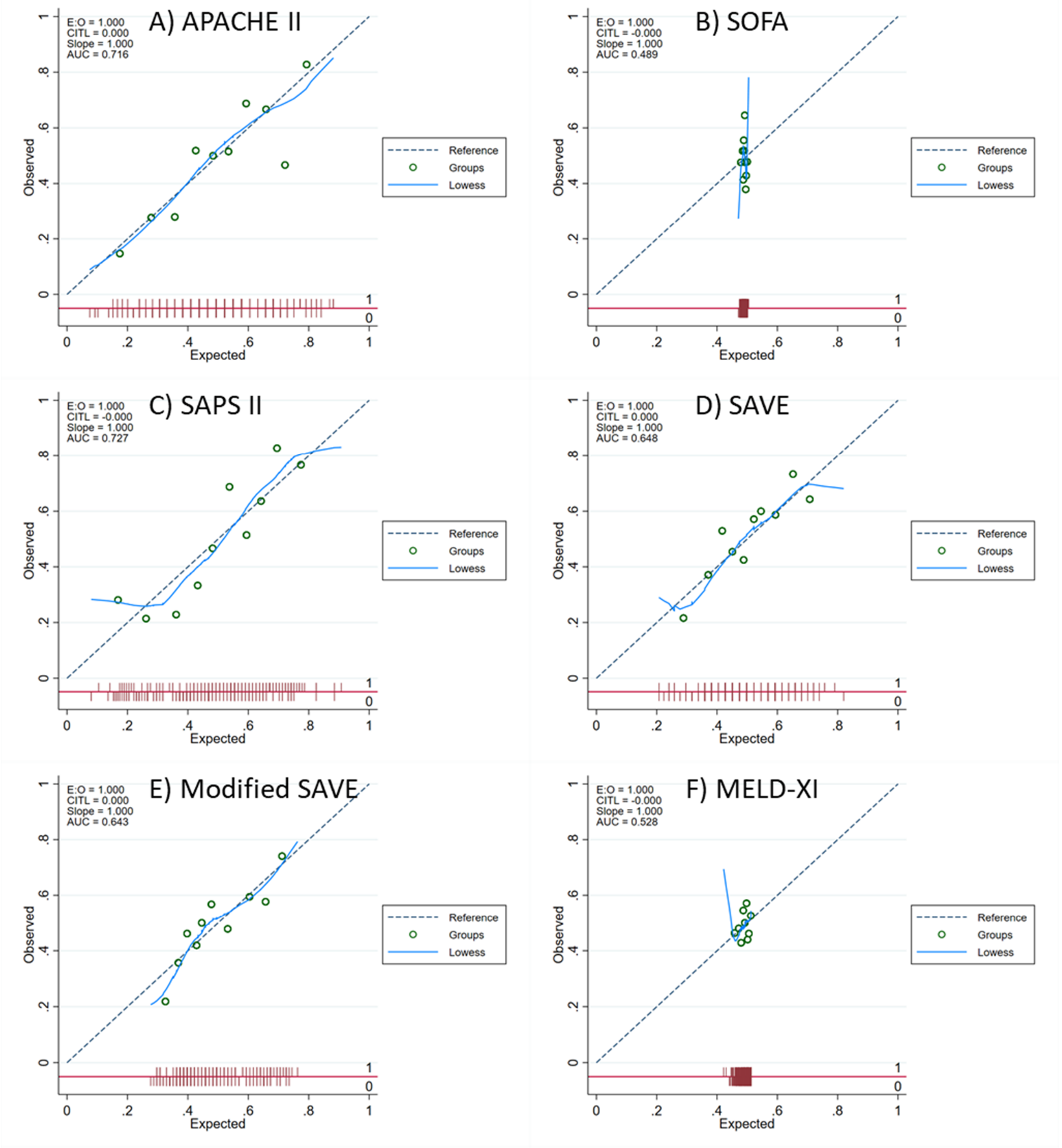

Discrimination was evaluated using AUC as given. Calibration was evaluated by lowess smoother curves and agreement in tenths of risk. Risk score distribution is provided in red below the x-axis.

Figure S2: Comparison of predictive performance for all VV ECMO scores.

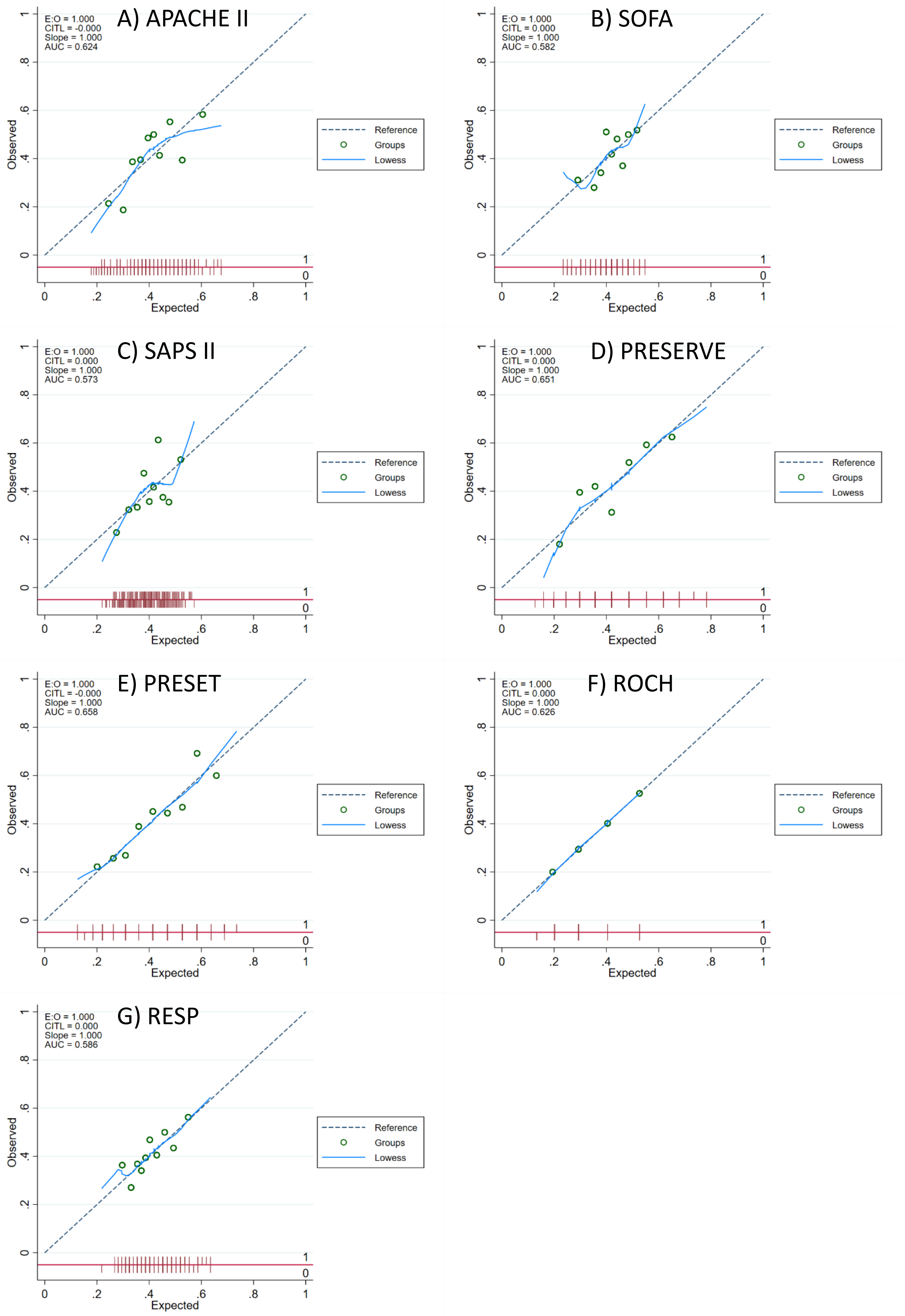

References

1 Knaus WA, Draper EA, Wagner DP, Zimmerman JE. APACHE II, A severity of disease classification system. *Critical care medicine* 1985; 13: 818–829.

2 Vincent JL, Moreno R, Takala J, Willatts S, Mendonça A de, Bruining H, Reinhart CK, Suter PM, Thijs LG. The SOFA (Sepsis-related Organ Failure Assessment) score to describe organ dysfunction/failure. On behalf of the Working Group on Sepsis-Related Problems of the European Society of Intensive Care Medicine. *Intensive care medicine* 1996; 22: 707–710.

3 Le Gall JR, Lemeshow S, Saulnier F. A new Simplified Acute Physiology Score (SAPS II) based on a European/North American multicenter study. *JAMA* 1993; 270: 2957–2963.

4 Schmidt M, Bailey M, Sheldrake J, Hodgson C, Aubron C, Rycus PT, Scheinkestel C, Cooper DJ, Brodie D, Pellegrino V, Combes A, Pilcher D. Predicting survival after extracorporeal membrane oxygenation for severe acute respiratory failure. The Respiratory Extracorporeal Membrane Oxygenation Survival Prediction (RESP) score. *American journal of respiratory and critical care medicine* 2014; 189: 1374–1382.

5 Schmidt M, Zogheib E, Rozé H, Repesse X, Lebreton G, Luyt C-E, Trouillet J-L, Bréchot N, Nieszkowska A, Dupont H, Ouattara A, Leprince P, Chastre J, Combes A. The PRESERVE mortality risk score and analysis of long-term outcomes after extracorporeal membrane oxygenation for severe acute respiratory distress syndrome. *Intensive care medicine* 2013; 39: 1704–1713.

6 Roch A, Hraiech S, Masson E, Grisoli D, Forel J-M, Boucekine M, Morera P, Guervilly C, Adda M, Dizier S, Toesca R, Collart F, Papazian L. Outcome of acute respiratory distress syndrome patients treated with extracorporeal membrane oxygenation and brought to a referral center. *Intensive care medicine* 2014; 40: 74–83.

7 Hilder M, Herbstreit F, Adamzik M, Beiderlinden M, Bürschen M, Peters J, Frey UH. Comparison of mortality prediction models in acute respiratory distress syndrome undergoing extracorporeal membrane oxygenation and development of a novel prediction score, The PREdiction of Survival on ECMO Therapy-Score (PRESET-Score). *Crit Care* 2017; 21: 788.

8 Heuman DM, Mihas AA, Habib A, Gilles HS, Stravitz RT, Sanyal AJ, Fisher RA. MELD-XI, A rational approach to "sickest first" liver transplantation in cirrhotic patients requiring anticoagulant therapy. *Liver transplantation : official publication of the American Association for the Study of Liver Diseases and the International Liver Transplantation Society* 2007; 13: 30–37.

9 Schmidt M, Burrell A, Roberts L, Bailey M, Sheldrake J, Rycus PT, Hodgson C, Scheinkestel C, Cooper DJ, Thiagarajan RR, Brodie D, Pellegrino V, Pilcher D. Predicting survival after ECMO for refractory cardiogenic shock, The survival after veno-arterial-ECMO (SAVE)-score. *Eur Heart J* 2015; 36: 2246–2256.

10 Chen W-C, Huang K-Y, Yao C-W, Wu C-F, Liang S-J, Li C-H, Tu C-Y, Chen H-J. The modified SAVE score, Predicting survival using urgent veno-arterial extracorporeal membrane oxygenation within 24 hours of arrival at the emergency department. *Crit Care* 2016; 20: R82.
